# The Multitrophic Effects of Climate Change and Glacier Retreat in Mountain Rivers

**DOI:** 10.1093/biosci/bix107

**Published:** 2017-09-20

**Authors:** Sarah C. Fell, Jonathan L. Carrivick, Lee E. Brown

**Keywords:** river ecosystem, food web, ecological network, alpine, biotic response

## Abstract

Climate change is driving the thinning and retreat of many glaciers globally. Reductions of ice-melt inputs to mountain rivers are changing their physicochemical characteristics and, in turn, aquatic communities. Glacier-fed rivers can serve as model systems for investigations of climate-change effects on ecosystems because of their strong atmospheric–cryospheric links, high biodiversity of multiple taxonomic groups, and significant conservation interest concerning endemic species. From a synthesis of existing knowledge, we develop a new conceptual understanding of how reducing glacier cover affects organisms spanning multiple trophic groups. Although the response of macroinvertebrates to glacier retreat has been well described, we show that there remains a relative paucity of information for biofilm, microinvertebrate, and vertebrate taxa. Enhanced understanding of whole river food webs will improve the prediction of river-ecosystem responses to deglaciation while offering the potential to identify and protect a wider range of sensitive and threatened species.

**The sustained dependency of human society on** hydrocarbons is predicted to increase global near-surface temperatures, particularly across the second half of the twenty-first century (IPCC 2013). Warming will be most pervasive in high-altitude (alpine) and -latitude (Arctic) regions and will be coupled with changing precipitation patterns (Gobiet et al. 2014). Significant climatic changes are already occurring within these environments, reducing the distribution, thickness, and permanency of ice sheets and driving the thinning and retreat of many mountain glaciers (Zemp et al. 2015). Continued retreat will alter the proportional contribution of ice melt, snow melt, and groundwater to proglacial mountain-river systems (Brown et al. 2007). Each of these water sources has a unique physicochemical signature and flow regime, which influences the assembly of river communities (Milner et al. 2009). Glacier retreat and loss will therefore alter the mosaic of aquatic habitats across glacier floodplains, threatening multiple endemic and rare species that often exist at their tolerance limits (Wrona et al. 2006, Brown et al. 2009, Jacobsen et al. 2012, Giersch et al. 2016). Wider alterations to the persistence, density, and distribution of species will combine to drive major biological reorganization of mountain-river ecosystems (Brown and Milner 2012, Jacobsen et al. 2012).

Most ecological research within proglacial-river systems has focused predominantly on populations or communities of specific taxonomic groups, particularly macroinvertebrates (figure 1). To move forward our understanding of how climate change and glacier retreat are reshaping whole aquatic ecosystems, there is a need to develop an integrated understanding spanning multiple taxonomic groups and trophic levels in glacier-fed rivers (e.g., bacteria, protists, fungi, algae, diatoms, invertebrates, mammals, amphibians, and fish; Clitherow et al. 2013). Individual- to population-level responses cannot always be extrapolated easily to predict links at a network level, given that emergent properties are characteristic of complex systems (Woodward et al. 2010). One means of integrating the interactions among species that are responding in unison to environmental change is within the context of food-web ecology.

**Figure 1.**
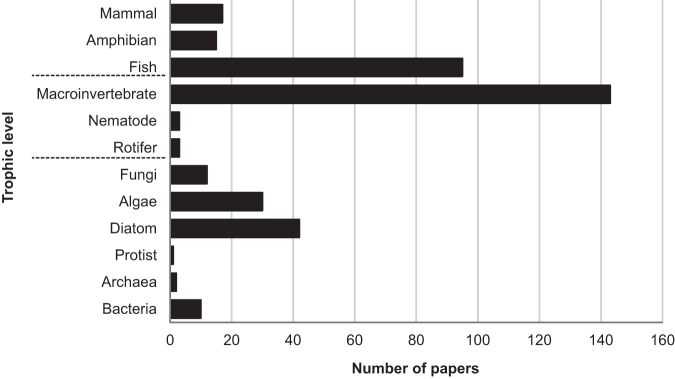
A summary of published literature regarding alpine-stream taxonomic groups and food webs in glacier-fed rivers from 1976 to 2017. The data extracted from the Web of Science (3 March 2017) were based on the following search criteria and mixtures thereof: taxa, alpine, river, stream, food web, and glacier. These search combinations identified research within glacier-fed rivers, even if they were not identified as such within publication titles.

This article provides an overview of global-scale patterns of glacier retreat and effects on mountain-river hydrological and physicochemical environments. We then synthesize the existing knowledge of how different groups of freshwater taxa (biofilm, invertebrates, and vertebrates) respond to glacier retreat, predominantly with a Northern Hemisphere focus because this is where most of the relevant research has been undertaken. This knowledge is then integrated within a new conceptual framework that considers simultaneous responses of biota to shrinking glaciers as part of multitrophic river ecosystems. This new multitaxonomic response framework is used subsequently to explore the consequences for how whole-river food webs can be expected to respond to ongoing glacier retreat. Such an approach is required to inform alpine conservation strategies by providing a holistic food-web context for the multiple cold-environment endemic species that are found in glacier-fed rivers around the world (e.g., Brown et al. 2009, Giersch et al. 2016). These often-rare species are potentially sensitive and vulnerable to climate change, and their successful conservation will require detailed consideration of their links within river assemblages. Because glacier-fed river systems will respond rapidly to climate change, any reassembly of food webs could help to identify structural and functional changes that could be monitored in running waters across other biogeographical regions (Woodward et al. 2010).

## Climate-change-induced glacier retreat in the twenty-first century

**Figure 2.**
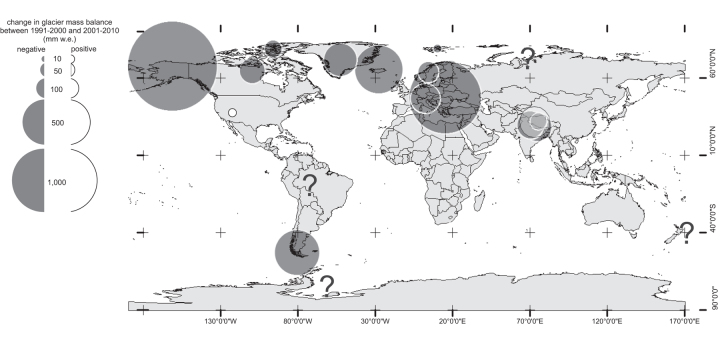
Global glacier mass balance alterations (1991–2000 and 2001–2010), adapted from Zemp and colleagues (2015). The question marks represent the absence of comparable data sets.

Arctic and alpine zones are experiencing pervasive increases in near-surface temperatures and altered patterns of precipitation (Gobiet et al. 2014), leading to the thinning and retreat of many glaciers (IPCC 2013). The magnitude of these changes is amplified within alpine regions as decreases in snow accumulation, earlier spring melt, and prolonged summer ice melt are altering surface albedo and lengthening the melt season. This increases energy absorption and sustains negative glacier mass balances (Gobiet et al. 2014). These positive feedback mechanisms are accelerating alpine glacier shrinkage in many regions (figure 2; Zemp et al. 2015). For large glaciers and ice sheets, such as those in parts of Iceland and Greenland, this ice melt will initially increase river discharge, scouring and exposing new channels as their margins recede, whereas smaller glaciers will see consistent reductions in runoff and eventually complete loss (Gobiet et al. 2014).

## Hydrology and physicochemistry of mountain rivers in a changing climate

**Figure 3.**
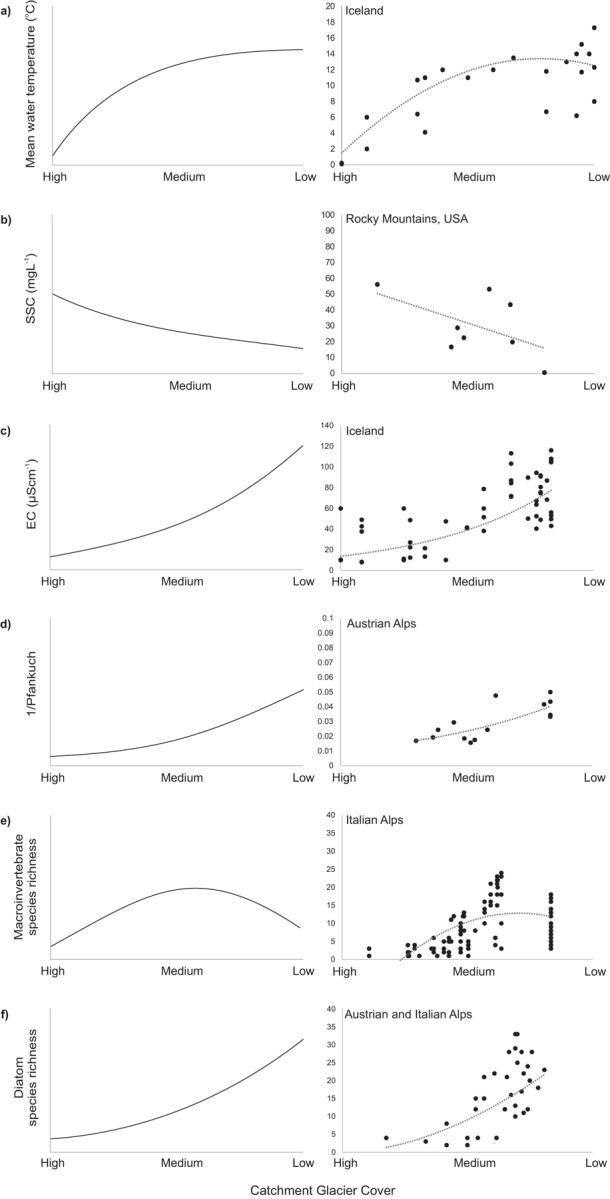
Theoretical predictions (left) and empirical data (right) for physicochemical-parameter responses to reducing catchment glacier cover across the Northern Hemisphere. Data adapted from (a) Gíslason and colleagues (2001), (b) Thompson and colleagues (2013), (c) Gíslason and colleagues (2001), (d) Khamis and colleagues (2016),  (e) Maiolini and colleagues (2001), and (f) Rott and colleagues (2006).

In addition to glacier ice melt, mountain rivers are supplied by runoff from snowpack ablation and groundwater. As was described by Brown and colleagues (2003), these water sources have distinct physicochemical compositions and discharge patterns. River reaches dominated by ice melt have significantly lower mean water temperatures, electrical conductivity, and channel stability and higher suspended-sediment concentrations and greater discharge fluctuations than groundwater reaches (figure 3). Ice-melt inputs reduce and groundwater influence increases with downstream distance from glacier margins, reflecting reducing catchment glacier cover (Brown et al. 2003). Temporal variability within water-source contributions is sustained by diel and seasonal ice-melt cycles, interannual alteration to snowpack accumulation, and intense storm events (Milner et al. 2009, Cauvy-Fraunié et al. 2013).

Mountain catchments are particularly vulnerable to climate change because glacier runoff significantly influences the source, rate, and timing of water directed to river networks (Brown et al. 2003, Jansson et al. 2003). Subsequently, prolonged glacier retreat will add further spatiotemporal variability to water-source patterns. Reducing ice-melt inputs will increase the proportional contribution of snow melt, rain, and groundwater (Brown et al. 2003), completely reworking the mosaic of channel environments present in glacier-fed river floodplains (Malard et al. 2006, Brown et al. 2015) and inducing significant reorganization of river biotic communities.

## Biotic responses to glacier retreat

Mountain-river ecosystems host a diverse range of taxa, which play varied trophic roles. Primary producers include bacteria, soft-bodied algae, and diatoms, whereas other bacteria, fungi, and protists play important roles as microbial decomposers and consumers of particulate and dissolved organic matter (Battin et al. 2016). Benthic rotifers feed on primary producers (bacteria and algae) and detritus alongside protozoans (Schmid-Araya 2000). Free-living nematodes also consume these groups, with some species predating rotifers and other meiofauna (Schmid-Araya 2000). Multiple trophic roles are also spanned by macroinvertebrates, with herbivores (grazers and scrapers) feeding on biofilm species, detritivorous shredders, collectors, and filter feeders consuming dead organic matter and predators selecting adult and larval invertebrates (Woodward 2009). Fish, primarily salmonids, are often the top predator of glacier-fed river systems, and although diet is species, life stage, and region specific, components include smaller fish, macroinvertebrates, and freshwater zooplankton (Sinnatamby et al. 2012). Some fish species also feed on the eggs of amphibians, which can be insectivorous or predatory (Arntzen et al. 2009, Kuzmin et al. 2009). Where present, semiaquatic mammals such as desman species (Talpidae) predate many trophic levels with diets spanning macrophytes, insects, fish, and amphibians (Biffi et al. 2016).

The diverse range of taxa within mountain rivers will respond simultaneously to water-source alterations imposed by glacier retreat (e.g., Brown and Milner 2012, Eisendle-Flöckner et al. 2013, Battin et al. 2016). It is important to review contemporary knowledge of these responses before attempting to understand holistically the reshaping of glacier-river ecosystems during deglaciation. Disparate literature considering biofilm, invertebrates, and vertebrates is collated here because despite a recent proliferation of studies considering particular trophic groups of alpine-river ecosystems, they are rarely considered collectively. Particular focus is given to less- studied groups that contribute to community response and encompass a broad range of endemism, rarity, and vulnerability to climate change.

### Bacteria and Archaea

**Figure 4.**
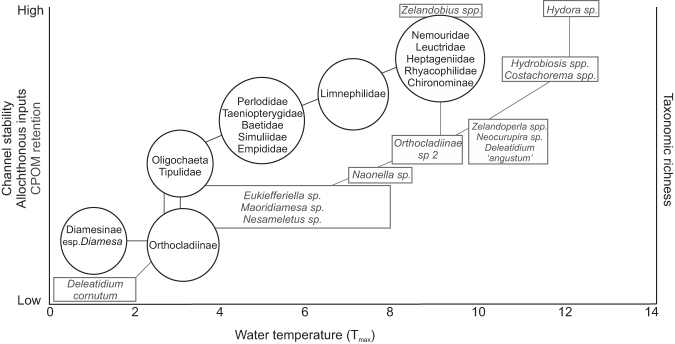
A composite figure based on the conceptual models of Milner and colleagues (2001; black text, circles) and Cadbury and colleagues (2011; gray text, squares) to illustrate the influence of water temperature and channel stability on macroinvertebrate community composition in Northern and Southern Hemisphere (New Zealand) sites, respectively.

Genetically diverse bacterial communities persist within alpine rivers, with Cyanobacteria (*Homeothrix, Clastidium*) dominating biofilm formation (Kawecka et al. 1971, Battin et al. 2001, Rott et al. 2006). Highly glacial rivers support *Bacteroidetes, Proteobacteria, Actionobacteria*, and *Nitrospira* (figure 4), with species that adhere to subglacial ice surfaces also contributing to river community composition following spring basal floods (Battin et al. 2001, Wilhelm et al. 2013). Archaea also inhabit glacier ice, entering stream biofilm communities during intensive melting events and reducing in density with increasing distance from the glacier terminus (Battin et al. 2001). Although reducing glacier influence increases bacterial biomass, Wilhelm and colleagues (2013) noted reductions in bacterial alpha and beta diversity as cold stenothermic species were replaced by generalist taxa (Freimann et al. 2013). However, this remains contested given that Battin and colleagues (2016) found alpha diversity to increase as ice melt exposed rock and soil habitats, which provide a greater diversity of microbe sources.

Glacial rivers are dominated by bacterial specialists, with groundwater species expressing greater metabolic redundancy and environmental plasticity (Freimann et al. 2013, Battin et al. 2016). This gradient of specialization is correlated with suspended sediment concentration and highly glacial sites host taxa adapted to reduced light penetration and greater abrasion (Peter and Sommaruga 2016). Glacier-margin habitats are also susceptible to spring-melt flood pulses, which constrain bacterial cell density through scouring, sheer stress, and fine-sediment abrasion of biofilm architecture (Blenkinsopp and Lock 1994). Reducing glacier influence may diminish this habitat heterogeneity, favoring a more generalist bacterial community (Freimann et al. 2013). Response to catchment-scale variability in electrical conductivity and pH can further influence local species dominance (Wilhelm et al. 2013, Battin et al. 2016).

### Fungi

Aquatic hyphomycetes dominate alpine-river fungal communities and are the principal microbial decomposers of allochthonous organic-matter inputs (Gessner and Robinson 2003). Specialist species are relatively unconstrained by cold temperatures and high suspended-sediment concentrations, with fungal biomass, taxonomic richness, sporulation rate, and diversity at glacial sites reduced but comparable to those of temperate rivers (Gessner and Robinson 2003). Decomposition rates are reduced 20%–60% at temperatures approaching 0 degrees Celsius, but it has been argued that this stems from a limited supply of organic matter rather than from physicochemical constraints on metabolism (Robinson et al. 1998). Reduced glacier influence may alter species dominance within fungal communities favoring those adapted to warmer waters, although this response will be mediated by local factors including nutrient supply and disturbance regime (Battin et al. 2016).

### Protists

The influence of glacier retreat on protists remains poorly understood (Rott et al. 2006, Battin et al. 2016). Eisendle-Flöckner and colleagues (2013) found a 35% reduction in catchment ice coverage in the Austrian Alps to double algal (minus diatom) abundance and increase protist abundance threefold, suggesting a stronger relationship with deglaciation than for other biofilm taxa. However, the absence of species-level identification and comparative studies hinders a more detailed analysis of this response. Low protist abundance where glacier influence is high (figure 4) may result from predation by meiofaunal invertebrates (Hakenkamp and Morin 2000), which can remain relatively abundant within cold conditions. Some protists can reach sizes that justify classification as meiofauna, and their preferential grazing of benthic bacteria and algae (Hakenkamp and Morin 2000) may limit the density of these taxa at less glacial sites.

### Soft-bodied algae

Filamentous algae, particularly *Hydrurus foetidus*, dominates high-altitude river biofilm (figure 4; Kawecka et al. 1971, Hieber et al. 2001). Although extensive catchment glaciation dramatically reduces algal species richness, density, and diversity, cold stenotherms adapt to variability in flow and nutrient pulses through alterations to cell physiology, life-cycle length, and preferential use of stable microhabitats (Kawecka et al. 1971, Rott et al. 2006). Sessile algae are influenced by seasonal variability in light availability, disturbance, and temperature (Kawecka 1971). Hieber and colleagues (2001) described the resulting proliferation of algal growth and chlorophyll production during spring and autumn: times of reduced suspended-sediment concentrations, increased solar radiation, and nutrient influx from snowpack melt. These blooms overlay a general increase in algal biomass with reducing glacier influence, a trend driven in part by groundwater blooms extending through summer (Lavandier and Décamps 1983, Rott et al. 2006). Glacial-river algae contribute significantly to the Red Lists of threatened algae across Europe (Ludwig and Schnittler 1996, Gesierich and Rott 2012).

### Diatoms

Unlike other algae, diatom species richness remains high until within very close proximity to glacier margins, and they form the principal food source of cold-adapted macroinvertebrates (Rott et al. 2006, Clitherow et al. 2013). As was shown by Gesierich and Rott (2012), *Hannaea arcus, Achnanthes* spp., *Diatoma* spp., and *Fragilaria* spp. consistently dominate glacial sites across Europe, North America, and the Himalayas (figure 4; Hieber et al. 2001, Antoniades and Douglas 2002, Rott et al. 2006). These pioneer species are small and nonmobile and resist abrasion in turbid glacial rivers through strong adhesion to substrates at the benthic interface (Hieber et al. 2001, Gesierich and Rott 2012). Antoniades and Douglas (2002) identified the specialist adaptions of *H. arcus* to cold waters and subsequent intolerance of groundwater. In contrast, species including *Diatoma mesodon* demonstrate greater environmental plasticity, occurring within subalpine and lower altitude rivers. Total diatom biomass increases as ice-melt inputs are reduced, but this proliferation may be constrained by herbivory, because grazing macroinvertebrates are more abundant within warmer, more stable rivers (Milner et al. 2009). The strong attachment capability of small epilithic diatoms may limit their consumption at glacial sites, increasing densities relative to other biofilm taxa (Gesierich and Rott 2012).

### Invertebrates

Although understanding of microinvertebrates (e.g., nematodes and rotifers) remains limited in comparison with that of macroinvertebrates (Thorp and Rogers 2011), Eisendle-Flöckner and colleagues (2013) studied these groups in detail across the Möll catchment, Austrian Alps, where they dominated the invertebrate community. Taxonomic richness increased with decreasing glacier influence, but density and abundance did not do so consistently, showing limited seasonal variability. This relationship requires further investigation, but elevated biomass despite harsh environmental conditions may be explained by the resilience traits of these pioneer taxa (Eisendle‐Flöckner et al. 2013, Robertson et al. 2015). At highly glacial sites, nematodes were more diverse but less abundant than rotifers (figure 4), and their maturity was constrained, suggesting a strong negative relationship with glacial influence (Eisendle‐Flöckner et al. 2013). Despite this, some meiofauna may be resilient to the high flows associated with large glaciers yet to reach peak retreat rates, because although Robertson and colleagues (2015) identified meiofaunal taxonomic abundance and richness to decline following an extreme rainfall event, they returned to preflood values for some rivers within 2 years. Protozoan responses to glacier retreat should be coupled with meiofaunal responses, because they feed on rotifers and are consumed by nematodes and microinvertebrates (Schmid-Araya 2000).

The relationship between alpine water sources and the macroinvertebrate component of river communities is well documented (Milner and Petts 1994, Castella et al. 2001, Jacobsen et al. 2012, Cauvy-Fraunié et al. 2015). Strong responses of macroinvertebrate alpha and beta diversity to glacier retreat have been linked to changes in water temperature and channel stability in many studies (Milner et al. 2001, Brown et al. 2007, Finn et al. 2013). Milner and Petts (1994) and then Milner and colleagues (2001) developed a conceptual model to include water temperature and channel stability as critical drivers of macroinvertebrate assemblage in glacially influenced rivers. This illustrated the reorganization of macroinvertebrate communities in response to reducing glacial influence (Milner and Petts 1994), embodying the individualistic concept (Gleason 1926) by attributing ecological communities to particular positions along natural gradients in response to their tolerances.

**Figure 5.**
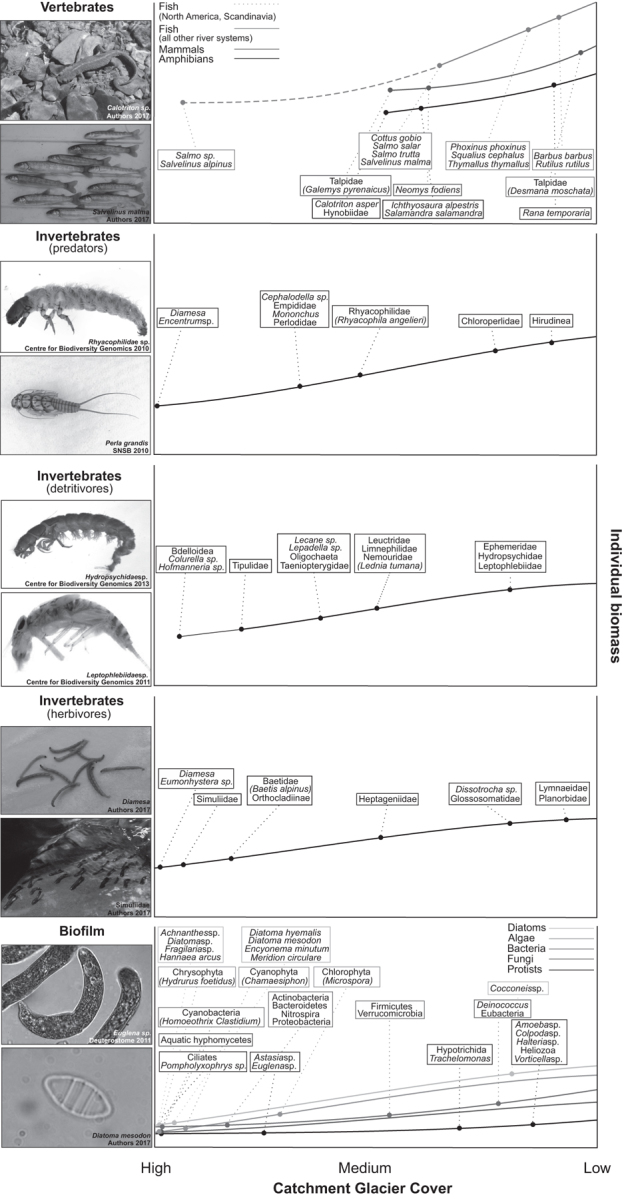
A conceptual model of the first appearance of key alpine-river taxa along a gradient of reducing catchment glacier cover across the Northern Hemisphere. The lines denote expected changes in individual biomass along the glacial gradient. Information regarding bacteria, protists, fungi, algae, diatoms, microinvertebrates, macroinvertebrates, mammals, amphibians, and fish is synthesized to predict biofilm, invertebrate, and vertebrate responses to reducing glacier influence.

Milner and colleagues’ (2001) revised model used information from the Arctic and Alpine Stream Ecosystem Research Program (AASER; figure 5). This conceptual synthesis incorporated information from a large number of European study sites and accounted for serial discontinuities by removing reliance on previous assumptions that confined low water temperature and channel stability to close glacial proximity (Milner et al. 2001, Milner 2016). Jacobsen and colleagues (2014) also identified temporal shifts in the distribution of glacier influence, with diurnal flood pulses altering downstream physicochemistry gradients and leading to the subsequent reorganization of macroinvertebrate communities. Figure 5 highlights the first occurrence of *Diamesa* in highly glacial sites and an increase in other macroinvertebrate groups—and therefore taxonomic richness, density, and biomass—with reducing glacier influence. Castella and colleagues (2001) demonstrated the pervasiveness of this pattern across Europe, identifying links between macroinvertebrate community structure, substrate stability, and water temperature in glacial rivers within five biogeographical regions across the Northern Hemisphere. Cadbury and colleagues (2011) later refined this model for Southern Hemisphere species, using data collected in alpine New Zealand (figure 5). Despite increases in macroinvertebrate richness and biomass, glacier retreat is reducing the beta and gamma diversity of alpine rivers globally (Brown et al. 2007, Jacobsen et al. 2012, Finn et al. 2013). This is driven by the extirpation of cold stenothermic species dependent on the physicochemical environment provided by ice-melt inputs (Giersch et al. 2016). This conceptual model has proven remarkably successful in explaining macroinvertebrate community structure patterns in many glacier-fed river environments (Milner 2016). It therefore has significant potential for being developed more widely to incorporate other taxonomic groups, something that to date has not been attempted.

### Mammals

Although few semiaquatic mammals inhabit alpine rivers, isolated species of Talpidae (desman) and Soricinae (water shrew) are found in localized populations (figure 4; Queiroz et al. 1996, Hutterer et al. 2016). The Iberian desman (*Galemys pyrenaicus*) shelter within the riparian vegetation and rocky banks of glacier-fed rivers, across the Pyrenean Region (Biffi et al. 2016). This species is indicative of low mean water temperatures and preferentially feeds within rapid, highly oxygenated riffles (Biffi et al. 2016). Their range is dictated by the presence of prey (*Trichoptera, Plecoptera, Ephemeroptera*) and the absence of predators, including American mink (*Neovison vison*; Biffi et al. 2016). The semiaquatic Eurasian water shrew (*Neomys fodiens*) mirrors the dependency of the Iberian desman for cold running waters, hunting insects, crustaceans, frogs, and fish below 2500 meters’ altitude (Hutterer et al. 2016). In contrast, the larger Russian desman (*Desmana moschata*) inhabits slow-flowing rivers and lakes of forested alpine floodplains (Ponomarev et al. 2015). Distributed across Russia, Belarus, Ukraine, and Kazakhstan, this species feeds omnivorously on macroinvertebrates, amphibians, fish, and plant detritus (Queiroz et al. 1996, Ponomarev et al. 2015). Both desman species are IUCN Red List vulnerable species, and reducing glacier influence may particularly threaten the Iberian desman (*G. pyrenaicus*), which is more heavily dependent on diminishing ice-melt reaches (Biffi et al. 2016).

### Amphibians

Salamandridae encompasses a number of amphibious species that rely on European mountain rivers. The common fire salamander (*Salamandra salamandra*) and alpine newt (*Ichthyosaura alpestris*) favor zones of reduced glacier influence, including alpine woodland rivers below 2500 meters (Kuzmin et al. 2009, Arntzen et al. 2009). In contrast, the Pyrenean brook newt (*Calotriton asper*) requires fast-flowing, highly oxygenated, cobbled river reaches for larval development (figure 4; Comas and Ribas 2015). The common frog (*Rana temporaria*) persists within mountain woodlands and meadows below 2300 meters, using rivers and lakes for larval development and overwintering in open water to avoid freezing conditions (Ludwig et al. 2015). Glacier retreat may alter the behavior of these species (e.g., abandonment of freeze-avoidance strategies) but not their persistence, because they also inhabit many nonglaciated mountain catchments (Ludwig et al. 2015). Glacier retreat therefore has the potential to create much more suitable river environments, aiding the spread of these large-bodied aquatic predators.

### Fish

Fish are often absent from highly glacial rivers, in which low water temperature constrains growth rates or fails to meet the optima required for particular life-cycle stages (Fleming 2005). Waterfalls and steep channel gradients may also limit their dispersal within mountain rivers. For many species, spawning is constrained in braided channel systems because of high suspended-sediment concentrations and an absence of stable pool environments (Milner et al. 2009). Despite this, large glacier-river systems in Alaska and the Rocky Mountains are able to support salmonid populations, because ice melt increases summer flow rates relative to clear-water tributaries, facilitating upstream migration and elevating nutrient and oxygen availability (figure 4; Dorava and Milner 2000). These systems also host slow-flowing side channels and pools suitable for rearing and juvenile overwintering, where high suspended-sediment conditions provide cover from aerial predators (Milner et al. 2011). Arctic charr (*Salvelinus alpinus*) have also colonized low-velocity proximal glacial streams in Arctic Canada and Norway, particularly where they are warmed by upstream glacial lakes (Witkowski et al. 2008, Sinnatamby et al. 2012).

A reduction of glacier ice melt could limit channel discharge, restricting salmonid migration and disconnecting channel marginal habitats. However, flow compensation from snow melt and groundwater sources could maintain high, less turbid discharge, aiding migration and spawning (Fleming 2005, Milner et al. 2009). Reducing glacier influence and subsequent increases in mean water temperatures may facilitate upstream dispersal of fish species as they track expansion of their thermal optima (Hari et al. 2005). Milner and colleagues (2011) found salmonids were able to rapidly recolonize proglacial channels within 10 years of their exposure from ice cover. This dispersal may be limited by anthropogenic barriers, including hydroelectric dams (Hari et al. 2005). Biological constraints include the comparatively reduced oxygen concentrations of warmer waters, novel predation pressures as communities reassemble, and the potential increase of temperature-dependent disease (Hari et al. 2005). Periods of high-velocity flow following intense ice-melt periods could act as physical barriers to fish dispersal within alpine streams (Sinnatamby et al. 2012).

## Synthesizing freshwater ecosystem responses to glacier retreat

Changes to alpine-river water sourcing driven by glacier retreat will clearly influence the processes of mountain-river ecosystem assembly for all trophic groups (Ilg and Castella 2006, Brown and Milner 2012). The discrete physicochemical and flow-regime characteristics of ice melt, snowpack melt, and groundwater act as strong abiotic filters in proglacial rivers, enabling only species with traits and behaviors adapted to these particular conditions to persist (Milner et al. 2001, Ilg and Castella 2006, Brown and Milner 2012). Although the majority of studies focusing on community assembly in glacier-fed river systems have worked with macroinvertebrates (figure 1; Ilg and Castella 2006, Brown and Milner 2012, Cauvy-Fraunié et al. 2015), evidence from other groups such as bacteria and protists also suggest a strong abiotic influence on assembly (Eisendle-Flöckner et al. 2013, Wilhelm et al. 2013). Species richness and density are reduced heavily in glacial rivers, but cold stenothermic species have adapted to persist within turbid conditions close to freezing (Wilhelm et al. 2013, Peter and Sommaruga 2016). In contrast, diatom and aquatic hyphomycetes communities are relatively unconstrained by harsh conditions (Gessner and Robinson 2003, Rott et al. 2006).

As environmental harshness declines with glacier retreat, there is expected to be a strong reduction in the influence of deterministic, abiotic control of community composition and subsequently a greater importance of stochastic and competition-driven assembly processes (Milner et al. 2011, Brown and Milner 2012). This effect has been noted for macroinvertebrates but may be altered with detailed consideration of assembly processes that consider interactions among multiple biological groups, many of which have been less well studied. Furthermore, the relative importance of biotic interactions and dispersal processes is still considered as a secondary effect in comparison with abiotic controls, despite recent observations of strong predation, omnivory, and cannibalism in glacially influenced rivers (Füreder et al. 2003, Clitherow et al. 2013, Khamis et al. 2015). Reductions in ice-melt inputs are already driving the reassembly of aquatic ecosystems, because species colonization is mediated by the efficacy of physiological and behavioral adaptions to imposed conditions (Ilg and Castella 2006, Milner et al. 2011, Brown and Milner 2012, Eisendle-Flöckner et al. 2013). There is therefore a pressing need to develop an understanding of the importance of whole-ecosystem interactions in these reassembly processes.

This literature synthesis has informed the development of a new conceptual framework that advances the widely accepted model of Milner and colleagues (2001) for macroinvertebrates (figure 5) to predict multitrophic responses to reducing glacier influence (figure 4). The first appearances of all biological groups considered in this synthesis are mapped simultaneously to a gradient of reducing catchment glacier cover. The purpose of this model is to inform holistic predictions of whole-alpine-river community reassembly and potential food-web restructuring in response to glacier retreat. It can thus serve as a focus for moving alpine-river science, conservation, and management beyond current paradigms that typically focus on single taxonomic groups.

This conceptual model is derived from the literature synthesis described above and therefore requires broader geographic validation and refinement to assess its general applicability, in a similar manner to which the model of Milner and Petts (1994) was revised by the coordinated European studies (AASER) described by Milner and colleagues (2001). At present, data are predominantly from the Northern Hemisphere, and although some studies demonstrate a global generality of macroinvertebrate responses to glacier retreat (e.g., Castella et al. 2001, Jacobsen et al. 2012), species assemblages require adjustment to reflect glacier-fed river ecosystems in the Southern Hemisphere (e.g., Cadbury et al. 2011, Jacobsen et al. 2014, Cauvy-Fraunié et al. 2015). The model has a temporal focus toward data collected during summer months and therefore might not adequately capture the seasonal biomass and diversity fluctuations of specific taxa (e.g., macroinvertebrates and nondiatom algae). Protist, nematode, and rotifer components in particular need much more work than other groups, because fewer studies were available to detail their responses (Eisendle-Flöckner et al. 2013, Robertson et al. 2015).

## Freshwater biodiversity responses to glacier retreat: Model application

From the multitrophic synthesis of population responses to glacier retreat, a number of general trends can be hypothesized that transcend most taxa. Figure 4 suggests both an increase in the biomass of most groups with reducing glacier influence, as well as a shift toward the introduction of more large-bodied predators (invertebrates, mammals, and amphibians) as glacier cover declines. This whole-food-web response is likely to underpin the decreasing part of the unimodal response of macroinvertebrate taxonomic richness and density at low glacier cover (Jacobsen et al. 2012). Although macroinvertebrate alpha diversity has been shown to peak at intermediate stages of glacier influence (Jacobsen et al. 2012, Brown et al. 2015), our synthesis suggests this response might not be generalizable to other groups. Vertebrates, for example, provide an exception to these trends, because desman species (*G. pyrenaicus*) and the Pyrenean brook newt (*C. asper*) preferentially occupy high-velocity mountain rivers in the Pyrénées, increasing their density and in turn biomass with reducing glacier influence (Comas and Ribas 2015, Biffi et al. 2016). Notably, they appear first at intermediate levels of glacier catchment cover (figure 4), in contrast to ubiquitous macroinvertebrates. They then continue to benefit from their improved habitat conditions, few if any larger predators, and abundant food sources as glaciers are lost.

For some biofilm groups (bacteria, archaea, algae, fungi, and protists) and meiofauna (rotifers and nematodes), the relationship between taxonomic richness and reducing glacier influence appears to be linear, resulting in increases in biomass with glacier retreat (figure 4). However, a relative lack of research focusing on the biomass of these groups along comprehensive gradients of glacier influence may mask a unimodal response as this linear trend is adjusted for groups including diatoms and protists (figure 4). Here, densities are relatively suppressed by intensified grazing pressures as meiofauna and macroinvertebrate densities increase (Hakenkamp and Morin 2000, Gesierich and Rott 2012). The additive influence of these varied, multiple taxa group responses needs to be explored in more detail, but the cumulative effect can be hypothesized as an increase in community biomass in response to glacier retreat, reaching some form of asymptote at low or no glacier cover. However, the presence of subsidies from terrestrial systems may alter the nature of the total biomass response, especially at low or no glacier cover, or in locations such as southeast Alaska and New Zealand, where glaciers often terminate in close proximity to forests.

Figure 4 illustrates a further trend that transcends most taxa: For the first appearing species, there is a transition from specialists to omnivorous generalists with higher trophic roles as glacier influence is reduced. Many cold-adapted species are recognized as threatened or endangered primarily because of climate-induced habitat contraction, which for fully aquatic taxa is compounded by limited dispersal opportunity when large mountain ranges isolate proglacial-river systems (Wrona et al. 2006, Brown et al. 2009, Giersch et al. 2016). These species include macroinvertebrates (*Baetis alpinus, Lednia tumana*, and *Rhyacophila angelieri*) but can be found at multiple trophic levels within diatoms (*D. mesodon* and *Diatoma hyemalis*), algae (Red List algae species), and vertebrates (*G. pyrenaicus* and *C. asper*; Ludwig and Schnittler 1996, Brown et al. 2007, Finn et al. 2013, Comas and Ribas 2015, Biffi et al. 2016, Giersch et al. 2016). The extirpation of macroinvertebrate stenotherms drives a reduction in gamma diversity across formally glacierized catchments (Jacobsen et al. 2012), a trend that will be exacerbated through the loss of cold stenotherms at additional trophic levels (figure 4).

## Food-web responses to glacier retreat

Although it is important to determine the responses of particular trophic groups to glacier retreat and climate change generally, constituent taxa will never be affected independently because of the diversity of feeding and competitive interactions. This means that extrapolating out to community or whole-ecosystem responses from population-level studies is likely to fail to capture the emergent properties that characterize complex ecological networks (Woodward et al. 2010). Our multitaxa response framework enables explorations of how whole mountain-river food webs will respond to deglaciation. Despite the significant contribution of network theory to freshwater science (Thompson et al. 2012), still only a handful of food webs have been constructed for proglacial-river systems. Most researchers have employed stable isotope analysis to map energy flow through benthic communities (Zah et al. 2001, Fellman et al. 2015), highlighting, for example, the importance of dissolved organic matter consumption by microbial groups supporting wider food webs. However, further comparison of food webs is required to compare different mountain water sources. More studies of species-level interactions using connectance food webs are crucial to the investigation of the direct and indirect cascades that will occur through entire river ecosystems with decreasing glacier influence (Clitherow et al. 2013).

**Figure 6.**
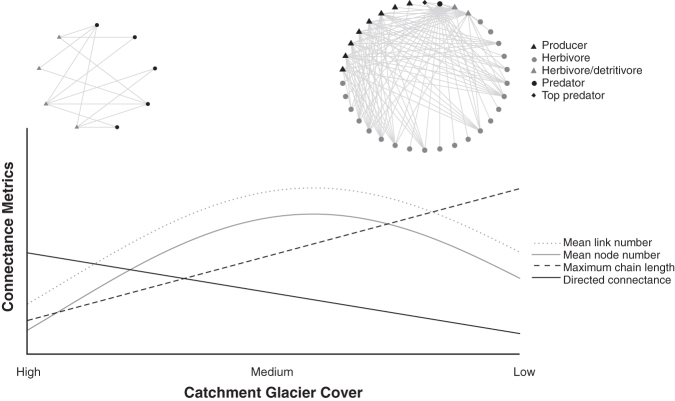
The predicted response of river food-web connectance metrics to reducing catchment glacier cover. Observed connectance food webs are displayed for sites of high (adapted from Clitherow et al. 2013) and low (adapted from Parker and Huryn 2006) glacier influence.

In a detailed mountain-river food-web study, Clitherow and colleagues (2013) used gut-content analysis to produce connectance and trivariate food webs for the river within 100 meters of the Ödenwinkelkees glacier, Austrian Alps (figure 6). Food webs were characterized by the highest connectance values (0.05–0.19) obtained for running waters, primarily because of generalist, opportunistic, and omnivorous macroinvertebrate feeding strategies in response to low primary production (Clitherow et al. 2013). Mean chain lengths were very short (2–2.27) because large predators were absent, given the cold-water temperature constraints on body size (Ilg and Castella 2006, Clitherow et al. 2013). This diminished the size structuring usually prevalent within freshwaters (Woodward 2009). The webs supported few nodes and links, reflecting the low densities and biomass of taxa illustrated in figure 4. Feeding links were predominantly between macroinvertebrates (Chironomidae, *Diamesa*) and both epilithic diatoms and detritus (Clitherow et al. 2013), as was noted in other studies (Zah et al. 2001, Füreder et al. 2003). Although unable to identify species-level connections, stable isotope analyses from other river systems have confirmed that the short mean chain lengths and low numbers of nodes and links in glacier-margin food webs result from macroinvertebrate feeding plasticity in response to an annually sustained autochthonous energy base (Fellman et al. 2015). Isotope methods can be particularly useful for detecting links not easily observed using gut-content approaches. For example, whereas Clitherow and colleagues (2013) inferred the importance of microbial subsidies of carbon and other nutrients in the Ödenwinkelkees food web, Fellman and colleagues (2015) were able to use ^14^C signatures to confirm that this was the case in other glacial rivers, where ancient carbon (hundreds of years old) released from the glacier was probably consumed by microbes and then assimilated by macroinvertebrates.

Parker and Huryn (2006) constructed connectance food webs for an Arctic mountain river lacking glacier influence (figure 6), which provides an indication of the changes that can be expected following glacier loss. A spring-fed food web supported slightly lower connectance (0.165–0.188) and longer mean chain lengths (2.98–3.10; Parker and Huryn 2006) than those documented in the study by Clitherow and colleagues (2013). They also encompassed on average a further 14.5 nodes and 62.5 links (Parker and Huryn 2006, Clitherow et al. 2013). Although an absolute comparison is affected by differences in sampling effort and taxonomy between the two studies, some general patterns can be deduced from this comparison. The Arctic food webs were influenced primarily by the addition of top predators, Dolly Varden trout (*Salvelinus malma*) and America dipper (*Cinclus mexicanus*), absent from the Austrian glacial food webs. In addition, Khamis and colleagues (2015) investigated the influence of increased macroinvertebrate predator abundance (*Perla grandis*) in spring-fed, *in situ* experimental mesocosm channels. Their results suggested that the future range expansion of this species (into streams that are currently highly glacial and therefore are unsuitable habitat) will increase trophic height (and therefore food-chain length) and body-size spectrums through invasion, intraguild predation, and interference competition (Khamis et al. 2015).

As prolonged glacier retreat will lead to ice-melt-influenced river reaches becoming dominated by groundwaters, it could be expected that their food webs will adopt the structural characteristics of spring-fed community networks (Lavandier and Décamps 1983). On the basis of this concept, figure 6 illustrates predicted changes to connectance food-web metrics with reducing catchment glacier cover. Construction of alpine-river food webs along gradients of glacier influence, using standardized methods and analysis techniques, is required to test these predictions further. In their absence, research investigating food-web responses to increasing water temperatures may be drawn on to explain this structural reassembly. Warming increases cold-water productivity, and more nutrient-rich waters may reduce directed connectivity by abating the requirement for flexible, opportunistic feeding strategies and diminishing the omnivory and cannibalism adopted to survive on a limited food supply (Lavandier and Décamps 1983, Friberg et al. 2009, Clitherow et al. 2013). Warmer waters will also reduce constraints on body mass and metabolic rates, hosting a greater abundance of larger individuals and supporting increased predator densities (Parker and Huryn 2006, Woodward et al. 2010). This, together with the upstream migration of ectothermic species following the expanding range of a particular life-stage thermal optimum, will increase food-chain lengths and the number of nodes (species) and links (Lavandier and Décamps 1983, Brown et al. 2007). The spectra of body sizes will also increase, strengthening size structuring, which can promote web stability (Woodward 2009).

Lavandier and Décamps (1983) identified connectance food webs to increase community abundance and the diversity of species along a gradient of increasing maximum water temperature within the snowmelt-fed Estaragne Basin, French Pyrénées. However, this proliferation may be constrained because novel species colonization can introduce increased or additional predation pressures, interference competition, and preferential suppression of particular prey (Parker and Huryn 2006, Wrona et al. 2006, Khamis et al. 2015). Nodes and link numbers may also be reduced by the extirpation of cold stenotherms through competition and habitat contraction (Hari et al. 2005, Wrona et al. 2006, Brown et al. 2007, Khamis et al. 2015).

Reducing glacier influence comprises more than an increase in river water temperature, with reducing suspended-sediment concentrations, discharge variability, and channel instability also influencing food-web structure (Parker and Huryn 2006, Dekar et al. 2009). Parker and Huryn (2006) found spring-fed rivers supporting these conditions to facilitate significantly increased bryophyte growth and persistence, in comparison with more unstable, dynamic channels. Bryophyte and epilithon communities provide structurally complex habitat refugia and food sources for macroinvertebrate larvae and meiofauna (Battin et al. 2016). Turbidity gradients also exert strong control on the density of bacteria (Peter and Sommaruga 2016). In turn, reduced disturbance regimes may provide a mechanism for increasing productivity, biomass, and species densities in mountain-river food webs, independently of mean water temperature.

Glacier retreat may further influence food-web structure indirectly, acting beyond water-source alteration. Prolonged retreat and the subsequent extension of the ice-free period will reduce the seasonality of river ecosystems, ensuring that production and reproduction become less confined to a short summer phase (Malard et al. 2006, Durant et al. 2007). This may increase energy availability to food webs, particularly through algal blooming, or uncouple the timing of consumer requirement from prey availability, reducing food-web links and potentially taxon survival (Durant et al. 2007). Significant ice loss could also lead to intermittency or cessation of flow (Ward et al. 1999). Periods of low or no flow could introduce trophic cascades of variable magnitude and may induce the compensatory reorganization of whole food-web cores, influencing all species within a community both directly and indirectly (Ledger et al. 2013, Lu et al. 2016).

## Conclusions

This synthesis article has presented a novel conceptual framework that collates the simultaneous responses of multitrophic river ecosystems to glacier retreat (figure 4). Knowledge of biofilm, invertebrate, and vertebrate responses from individual studies can inform holistic predictions of the rapid reshaping of mountain-river ecosystems in response to climate change. Although the responses of certain taxa remain poorly resolved, general predictions from the best-studied groups can nevertheless guide understanding of food-web responses to deglaciation. Glacial reaches are expected to shift over time to develop the structural characteristics of contemporary rain- and groundwater-fed food webs (figure 6). Increases in mean water temperature and channel stability will drive reduced directed connectance and increased mean chain lengths, predator densities, energy availability, size structuring, and the relative contribution of biotic (particularly competition) influences on community assembly (Parker and Huryn 2006, Woodward et al. 2010, Brown and Milner 2012). Mean numbers of nodes and links may initially increase as more and larger generalist species proliferate, broadening the spectrum of body sizes (Friberg et al. 2009). However, the potential extinction of some cold specialist species due to novel predation pressure and habitat reduction may constrain these metrics (Hari et al. 2005, Wrona et al. 2006, Brown et al. 2007, Giersch et al. 2016). Ice melt could induce further structural reorganization as reduced seasonality, periodic drought, and potential flow cessation alter energy-production rates and network stability (Durant et al. 2007, Lu et al. 2016). The scientific community places “high confidence” in the accelerating and pervasive nature of worldwide glacial shrinkage (IPCC 2013, p. 4). The associated threats to mountain-river biodiversity (Jacobsen et al. 2012) highlight the urgent need for an improved understanding of these predicted responses.

Future research should investigate protist, nematode, rotifer, virus, and protozoa communities in much more detail, given that less is known regarding the response of these groups to glacier retreat and especially their role within food webs, which often reveal vertebrate–macroinvertebrate–algal links. Protist species should receive particular focus because their densities appear to be more sensitive to deglaciation than those of other freshwater taxa (Eisendle‐Flöckner et al. 2013). Species whose density and richness are influenced minimally by glacial conditions (e.g., aquatic hyphomycetes, diatoms, and rotifers) should be highlighted as potential conservation priorities, because intensive specialization may increase their vulnerability to water-source alterations (Wrona et al. 2006). Comparative studies are required to determine food-web structure both at the extremes of glacier influence and along a quantified spectrum of intermediary stages to investigate the ecosystem-level effects of glacier retreat (Clitherow et al. 2013). There is also requirement for more winter sampling (Brown et al. 2015) to determine the influence of seasonal variability in biomass (macroinvertebrate, nondiatom algae) on ecosystem structure and food-web dynamics.

Despite the need for further validation, our new conceptual framework offers immediate potential to inform conservation management strategies by highlighting that cold stenothermic species are found beyond the macroinvertebrate component of river communities (e.g., algae, diatoms, and vertebrates) and that other taxonomic groups can serve as differential indicators of climate change in mountain-river systems. It also holds value beyond alpine rivers because the reorganization of communities within glaciated headwaters will intrinsically influence the species pools available to colonize downstream reaches, potentially reshaping their assembly processes (Brown and Milner 2012). The often-strong deterministic effects of habitat on alpine-river biological communities mean that they are valuable model systems for understanding ecosystem responses to environmental change. Coupled with the potential for species interactions to be investigated and manipulated easily and comprehensively (e.g., Khamis et al. 2015, Cauvy-Fraunié et al. 2016), glacier-fed rivers offer significant potential to inform mechanistic predictions in other river systems that will be modified by environmental change.
